# The Mysteries of Chromosome Evolution in Gibbons: Methylation Is a Prime Suspect

**DOI:** 10.1371/journal.pgen.1000501

**Published:** 2009-06-26

**Authors:** Judith D. Brown, Rachel J. O'Neill

**Affiliations:** Department of Molecular and Cell Biology, University of Connecticut, Storrs, Connecticut, United States of America; University of Cambridge, United Kingdom

Dobzhansky and Sturtevant provided the first view of the molecular basis of species identity in their 1938 seminal study classifying the chromosome rearrangements that distinguish two *Drosophila* species [1]. Decades of study of genome architecture from an evolutionary perspective then followed, enriching our knowledge of developmental genetics, gene regulation, human genetic disorders, and cancer, while greatly contributing to the neo-Darwinian view of the divergence of species.

The view that has emerged over the last decade, with a sharp acceleration since the publication of the human genome sequence, is of a fluid genomic landscape that is dotted with evidence of both large- and fine-scale chromosome rearrangements. What has remained a mystery are the mechanisms responsible for chromosome rearrangements that karyotypically define species. In this issue of *PLoS Genetics*, Lucia Carbone et al. [2] use the northern white-cheeked gibbon (*Nomascus leucogenys leucogenys*) to address a fascinating problem in evolutionary biology: why are some groups of organisms characterized by a high frequency of chromosome change while others are karyotypically stable?

Gibbons are members of the Hominoidea superfamily, which includes humans and great apes, but they are unique among Hominoidea, and indeed rare among mammals, in having experienced an extraordinarily high rate of karyotypic change [2]. Carrying a remarkable number of lineage-specific breaks of synteny, the four genera of gibbons separated from their common hominoid ancestor with humans between 15 and 20 million years ago [3]. Gibbons carry a broad array of chromosome rearrangements, including pericentric and paracentric inversions, fissions, fusions, and Robertsonian and reciprocal translocations, placing this group of endangered mammals among the most karyotypically diverse within primates.

Building on their previous work defining the synteny map for the northern white-cheeked gibbon with respect to its human cousin, these authors used a comparative genomics approach to analyze sequences spanning breaks of synteny for repeat distribution and genomic signatures that would lend some insight into the mechanism of interchromosomal rearrangement. Corroborating data from other studies on a smaller set of gibbon breakpoints [4],[5], this analysis of 57 breakpoints found a correlation between segmental duplications and breaks of synteny. While there is clearly a tight association between segmental duplications and chromosomal breaks in many primate lineages (including humans), it is apparent from these studies that many segmental duplications in gibbons are specific to the gibbon lineage and are thus not a contributor to the initial cascade of events responsible for the rearrangements themselves, but rather are a result of the double-strand break events at these rearrangement sites [2],[4],[5].

Rather than simply quantifying the repeat classes at the gibbon-specific breaks of synteny, Carbone et al. took this study one step further by asking whether the epigenetic signatures of specific repeat classes may be an important distinguishing feature in highly divergent genomes. Previous work has shown that gibbon Alu elements are more active than their human counterparts [6]. Taken with the observation from Carbone et al. that the Alu elements found at gibbon breaks of synteny carry a higher CpG content, the control of mobile element activity by DNA methylation stands out as a potential epigenetic signature at these breakpoints.

The epigenetic alteration of genomic sequences by DNA methylation is appreciated as a major regulatory force in the evolution of genome structure and expression, and is known as a potent regulator of mobile DNA activity. Through bisulfite sequence analysis, the authors show that the gibbon Alus are undermethylated compared to their human orthologues. The authors suggest these epigenetic differences between human and gibbon as a possible mechanism to account for the disparity in the number of chromosome rearrangements between the gibbons and old world primates.

The proposal that mobile DNA itself participates in DNA rearrangement is not new to biology. Mobile elements, such as transposons and retrotransposons, were first implicated in DNA rearrangements in studies of maize by McClintock [7]. Their mobility is known to alter chromosome structure as well as gene expression, and may promote the genetic variability necessary for rapid evolution. Others have proposed that chromosomal rearrangements can promote reproductive isolation between species and may lead to rapid speciation [8],[9]. Hybridization between these two populations could then lead to mobilization of transposable elements that could cause the dysgenesis of hybrids.

The novelty in this study is that there is hypomethylation of the gibbon Alus at evolutionary breakpoints, and thus the epigenetic architecture of these regions may have facilitated the rearrangements in the gibbon karyotype. The lower levels of methylation in these repeats may lead to an open chromatin configuration that increases the opportunity for double-strand breakage and repair mechanisms such as intrachromosomal non-allelic homologous recombination and non-homologous end joining (Figure 1). However, many of the gibbon breakpoints do not carry a signature (such as microhomology or Alu-Alu recombination events) that easily implicates any particular mechanism of rearrangement. Thus it is intriguing to consider the possibility that the epigenetic state of specific elements may have been disrupted at some point during the evolution of this gibbon species, which in turn increased the frequency for such elements to participate in rearrangement.

**Figure 1:**
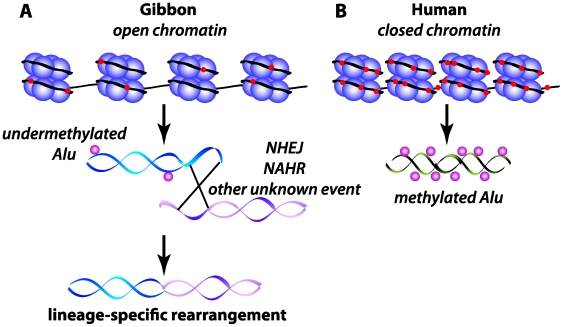
Schematic of epigenetic state of Alu elements at gibbon and human orthologous evolutionary breakpoints. (A) Gibbon breakpoint region containing an undermethylated Alu, resulting in open chromatin, and (B) human orthologous region containing a methylated Alu and closed chromatin. DNA (black) is wrapped around nucleosomes (purple) showing relative DNA methylation levels (red). The possible rearrangement mechanisms are indicated on the affected DNA molecules in the gibbon. NAHR, non-allelic homologous recombination; NHEJ, non-homologous end joining.

McClintock first implicated transposable elements in the speciation process when she stated that “species crosses are…a potent source of genomic modification” and that “major restructuring of chromosome components may arise in a hybrid” [10]. She added species crosses to the growing list of genomic stresses that could cause the activation of mobile elements. Given the suggestion that gibbons may have experienced hybridization events sometime in the last 15 million years [11], hybridization-induced perturbation of mobile element methylation and stability [12],[13] may be one process through which these mobile elements participate in genome shuffling [2].

Exciting advances in sequencing technology will now afford full genome-scale methylation studies (i.e., characterization of the full methylome) that can offer insight into the diversity of elements that may be differentially methylated between gibbons and humans, and whether Alus are the sole target. Additionally, testing for a similar association between mobile DNA and methylation state at breaks of synteny in other species groups that have experienced rapid karyotypic change (such as mice, dogs, horses, and kangaroos) are exciting areas of future work that may finally shed light on the mechanisms responsible for the chromosome diversity observed in a broad range of species.

## References

[1] Dobzhansky T, Sturtevant A (1938). Inversions in the chromosomes of *Drosophila pseudoobscura*.. Genetics.

[2] Carbone L, Harris RA, Vessere GM, Mootnick AR, Humphray S (2009). Evolutionary breakpoints in the gibbon suggest association between cytosine methylation and karyotype evolution.. PLoS Genet.

[3] Goodman M (1999). The genomic record of Humankind's evolutionary roots.. Am J Hum Genet.

[4] Carbone L, Vessere GM, ten Hallers BF, Zhu B, Osoegawa K (2006). A high-resolution map of synteny disruptions in gibbon and human genomes.. PLoS Genet.

[5] Girirajan S, Chen L, Graves T, Marques-Bonet T, Ventura M (2009). Sequencing human-gibbon breakpoints of synteny reveals mosaic new insertions at rearrangement sites.. Genome Res.

[6] Nakayama K, Ishida T (2006). Alu-mediated 100-kb deletion in the primate genome: The loss of the agouti signaling protein gene in the lesser apes.. Genome Res.

[7] McClintock B (1987). The discovery and characterization of transposable elements.

[8] Fontdevila A (1992). Genetic instability and rapid speciation: Are they coupled?. Genetica.

[9] White M (1978). Modes of speciation.

[10] McClintock B (1984). The significance of responses of the genome to challenge.. Science.

[11] Arnold ML, Meyer A (2006). Natural hybridization in primates: One evolutionary mechanism.. Zoology (Jena).

[12] Brown JD, Golden D, O'Neill RJ (2008). Methylation perturbations in retroelements within the genome of a Mus interspecific hybrid correlate with double minute chromosome formation.. Genomics.

[13] O'Neill RJ, O'Neill MJ, Graves JA (1998). Undermethylation associated with retroelement activation and chromosome remodelling in an interspecific mammalian hybrid.. Nature.

